# Mobile authentication of copy detection patterns

**DOI:** 10.1186/s13635-023-00140-5

**Published:** 2023-06-06

**Authors:** Olga Taran, Joakim Tutt, Taras Holotyak, Roman Chaban, Slavi Bonev, Slava Voloshynovskiy

**Affiliations:** Stochastic Information Processing Group, Department of Computer Science, University of Geneva, 7 Route de Drize, 1227 Carouge, Switzerland

**Keywords:** Authentication, Copy detection patterns, Copy fakes, Multi-class classification, One-class classification

## Abstract

In the recent years, the copy detection patterns (CDP) attracted a lot of attention as a link between the physical and digital worlds, which is of great interest for the internet of things and brand protection applications. However, the security of CDP in terms of their reproducibility by unauthorized parties or clonability remains largely unexplored. In this respect, this paper addresses a problem of anti-counterfeiting of physical objects and aims at investigating the authentication aspects and the resistances to illegal copying of the modern CDP from machine learning perspectives. A special attention is paid to a reliable authentication under the real-life verification conditions when the codes are printed on an industrial printer and enrolled via modern mobile phones under regular light conditions. The theoretical and empirical investigation of authentication aspects of CDP is performed with respect to four types of copy fakes from the point of view of (i) multi-class supervised classification as a baseline approach and (ii) one-class classification as a real-life application case. The obtained results show that the modern machine-learning approaches and the technical capacities of modern mobile phones allow to reliably authenticate CDP on end-user mobile phones under the considered classes of fakes.

## Introduction

In the modern world of globally distributed economy, it is extremely challenging to ensure a proper production, shipment, trade distribution, consumption, and recycling of various products and goods of physical world. These products and goods range from everyday food to some luxury objects and art. Creation of digital twins of these objects with appropriate track and trace infrastructures complemented by cryptographic tools like blockchain represents an attractive option. However, it is very important to provide a robust, secure, and unclonable link between a physical object and its digital representation in centralized or distributed databases. This link might be implemented via overt channels, like personalized codes reproduced on products either directly or in a form of coded symbologies like 1D and 2D codes or covert channels, like invisible digital watermarks embedded in images or text or printed by special invisible inks. However, many codes of this group are easily copied or can be regenerated. Thus, there is a great need in unclonable modalities that can be easily integrated with the printable codes. This necessity triggered the appearance and growing popularity of Printable Graphical Codes (PGC). During the last decade, the PGC attracted many industrial players and governmental organizations. One of the most popular nowadays type of PGC is a union of traditional 2D codes and *copy detection patterns* (CDP) [1–4].

**Fig. 1:**
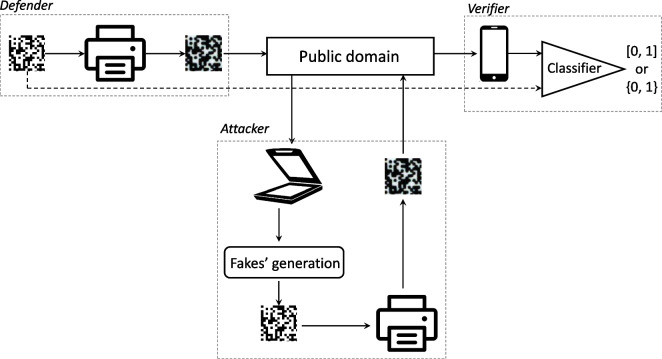
General scheme of the CDP life cycle starts from the generation of the digital templates by the defender and their following printing. The produced codes go to the public domain. An attacker has an access to the publicly available printed codes and can produce different type of fakes that are then also distributed in the public domain. A verifier should digitize the printed codes from the public domain and validate them via some classifier. As it is shown by the dashed line, the validation might be produced with or without taking the digital templates into account. For the defender-verifier pair, the main goal is to minimize the probability of error. In contrast, the attacker aims at maximizing the probability of error

General scheme of the CDP life cycle is shown in Fig. 1. The CDP security is based on a so-called information loss principle: each time the code is printed or scanned, some information about the original digital template is inevitably lost. In the case of printable codes, the information loss principle is based on physical phenomena of random interaction between the ink or toner with a substrate [5]. As a result, any dot undergoes a complex unpredictable modification and changes its shape accordingly to a dot gain effect. Generally, the black dot increases in its size. A white hole on a black background accordingly decreases its area due to the dot gain of nearest black dot surround.

In the case of image acquisition, the information loss principle refers to a loss of image quality due to various factors that include variability of illumination, finite and discrete nature of sampling in CCD/CMOS sensors, non-linearity in sensor sensitivity, sensor noise and various sensor defects, etc. All together, the enrolled image is characterized by some variability that degrades the quality of image in terms of its correspondence to the original digital template from which the code was printed.

Nowadays, there exists a big variety of different approaches aiming to combine CDP and widely used traditional 2D codes. Without pretending to be exhaustive in the presented overview, some of the most representative approaches are mentioned below.

**Fig. 2:**
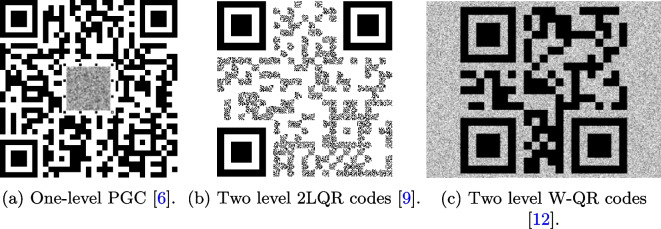
Examples of different types of modern PGC with CDP modulations

In general, it is possible to distinguish the standard one-level PGC and more advanced multi-level PGC. Examples of these codes are given in Fig. 2. The one-level PGC is shown in Fig. 2a. According to the presented design, a CDP central part is inserted into a structure of 2D QR-code [6]. Originally, the multi-level PGC aimed at increasing the storage capacity of the regular PGC [7]. Recently, the multi-level PGC are considered as a tool to increase the security of standard PGC. Without loss of generality, it is possible to identify the multi-level PGC with a modulation of the main black symbols as shown in Fig. 2b and a background modulation as illustrated in Fig. 2c.

The most well known multi-level PGC of the first type are so-called two level QR (2LQR) codes proposed in [8, 9], where the standard black modules are substituted by special modulated patterns. The general principles of modulation of multi-level codes were initially considered and theoretically analyzed in [7]. The public level of this code is read as normal standard QR code. The texture patterns are chosen to be sensitive to the print and scan process. At the same time, the modulation pattern can carry out private message. Furthermore, the idea of 2LQR was extended in [10] by the use of different encrypting strategies. The anti-counterfeiting performance of these codes was mainly tested based on desktop printers and scanners [8, 9]. Thus, there is a great interest in validation of these codes under the industrial printing and mobile phone authentication.

The second type of multi-level PGC is so-called W-QR codes proposed in [11], where the authors substitute the background of a standard QR code by a specific random texture. The embedded texture does not affect the readability of the standard code, but it should be sensitive to the print and scan process in such a way to give a possibility to authenticate the original code from the counterpart. The authors propose a particular random textured pattern, which has a stable statistical behavior. Thus, the attacker targets to estimate the parameters of the used textured pattern.

Despite the differences in ways how the traditional QR codes and CDP are combined, in general case, the authentication of digital artwork based on the CDP is done by comparing the reference template with the printed version scanned using a scanner or camera of mobile phone. As a reference template, there can be used either a digital template or enrolled printed version of the same artwork. The comparison can be done in different ways either in the spatial or frequency domain using a correlation, distance metrics, or a combined score of different features, etc., [2, 12]. Alternatively, one can also envision an authentication in a transform domain using latent space of pretrained classifiers or auto-encoders [13].

Despite a great interest, the robustness of CDP, used in PGC, to the copy attacks remains a little studied problem. Therefore, the current work is dedicated to the investigation of the authentication aspects of CDP under industrial settings from the perspective of modern machine learning.

The main contributions of this paper are:

- We provide the extended representation of production and enrollment procedures and settings of the Indigo mobile dataset of CDP created under the regular industrial settings and briefly presented in [14].
- We provide an extention of the multi-class supervised classification results presented in [14]. Namely, in addition to the supervised classifier trained in the binary (or two classes) setup with respect to the different types of the fakes, we provide new results of the performance of supervised classifier trained in three and five classes classification setups.
- We investigated the authentication aspects of the CDP from the perspective of one-class classification in the spatial domain with respect to the different type of reference codes: the digital templates and the physical references.
- For the one-class classification in the deep processing domain, we provide more detailed mathematical explanation of the model under investigation.
- In addition to the five basic scenarios of the one-class classification based on the one-class SVM, we provide more deep investigation of the problem under investigation with respect to the Hamming distance decision criteria. Also, we provide more detailed analysis of the latent space of the deep models under investigation.
- Finally, we investigate the complexity of the main models under investigation.

### Notation

We use the following notations: ${\textbf {t}} \in \{0, 1\}^{m \times m}$ denotes an original digital template; ${\textbf {x}} \in \mathbb {R}^{m \times m}$ corresponds to an original printed code, while ${\textbf {f}} \in \mathbb {R}^{m \times m}$ is used to denote a printed fake code; ${\textbf {y}} \in \mathbb {R}^{m \times m}$ stands for a probe that might be either original or fake. $p_t({\textbf {t}})$ and $p_{\mathcal {D}}({\textbf {x}})$ correspond to empirical data distributions of the digital templates and original printed codes, respectively. The discriminators corresponding to Kullback-Leibler divergences are denoted as $\mathcal {D}_{\textrm{x}}$, where the subscript indicates the space to which this discriminator is applied to.

## Datasets

### State-of-the-art datasets

The majority of the research experiments in the domain of CDP are performed either on synthetic data or on small private datasets. The production of datasets of real CDP is a very time consuming and quite costly process. It requires the printing and acquisition of the original CDP, the production and acquisition of fakes preferably on the equipment close to the industrial one.

Up to our best knowledge, there are only few publicly available datasets that were created to investigate the clonability aspects of CDP:

1. The DP0E [15] and its extension DP1E & DP1C [13] are the datasets of real and counterfeited CDP based on *DataMatrix* modulation [16] printed at resolution 1200 dpi with four printers: two laser (a) Samsung Xpress 430 and (b) Lexmark CS310 and two Inkjet (c) Canon PIXMA iP7200 and (d) HP OfficeJet Pro 8210. The enrollmen was performed by using the high resolution scanners at resolution 1200 ppi: Canon 9000F and Epson V850 Pro. The DP1E & DP1C dataset contains 6528 codes produced from 384 digital templates with symbol size $6 \times 6$ elements, with 3072 printed original codes and 3072 fake codes printed on the same printers as original codes.
2. The CSGC dataset [17] consists of 3800 codes produced from 950 digital templates with symbol size $1 \times 1$ elements and 2850 original codes printed on the Xerox Phaser 6500 laser at resolution 600 dpi and scanned by the Epson V850 Pro scanner under three resolutions: 2400 ppi, 4800 ppi, and 9600 ppi.
3. Indigo mobile dataset [14] contains the CDP printed on the industrial printer HP Indigo 5500 DS at resolution 812 dpi. This dataset was created to investigate the authentication capabilities of CDP under conditions closer to the real-life environment. In this respect, instead of high quality scanners, the printed codes were enrolled by a mobile phone *iPhone XS* under regular room light conditions. The dataset contains 300 digital templates with symbol size $5 \times 5$ elements, 300 printed original codes, and 1200 typical copy fake codes.

As an example of the real-life scenario, the Indigo mobile dataset presents a particular interest for the detailed practical investigation.

### Indigo mobile dataset

**Fig. 3:**
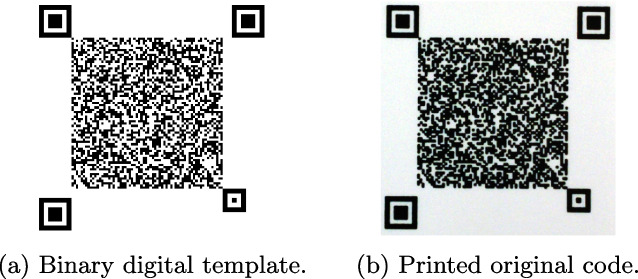
Examples of **a** a binary digital template used for printing and **b** the printed original code from the Indigo mobile dataset enrolled by the mobile phone

**Fig. 4:**
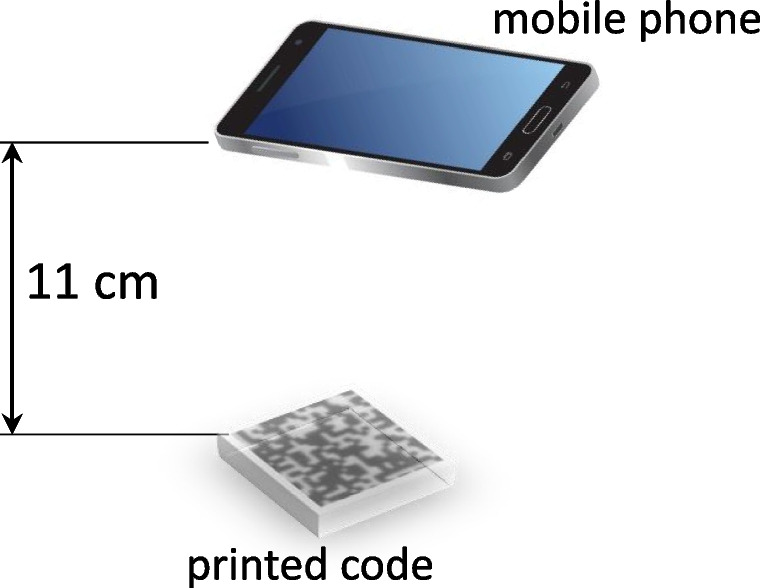
The schematic representation of the mobile phone acquisition setup

**Fig. 5:**
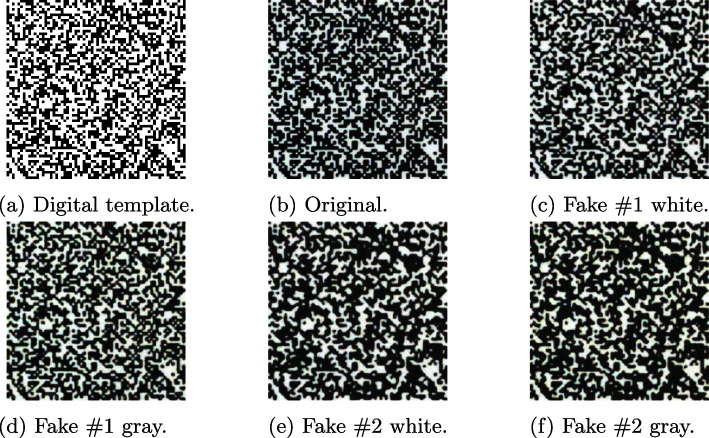
Examples of original and fake codes with symbol size $5 \times 5$ elements taken by a mobile phone from the Indigo mobile dataset

Indigo mobile dataset includes 300 distinct digital *DataMatrix* templates ${\textbf {t}} \in \{0, 1\}^{330 \times 330}$ with the symbols of size $5 \times 5$ elements^1^. An example of the digital template is given in Fig. 3a. The digital templates consist of the central CDP and four synchro-markers that allow to make an accurate synchronization and cropping of the code of interest. To simulate the real-life scenario, the generated digital templates were printed on the industrial printer *HP Indigo 5500 DS* at the resolution 812 dpi^2^. The acquisition of the printed codes is performed under regular room light using mobile phone *iPhone XS* (12 Mpixels) under the automatic photo shooting settings in Lightroom application^3^. The mobile phone is held parallel to the printed code at height 11 cm as schematically shown in Fig. 4. The photos are taken in DNG format to avoid built-in mobile phone image post-processing. An example of obtained photo is shown in Fig. 3b. The following cropping of the code is performed in an automatic way by applying a geometrical synchronization with four squared synchro-markers. Finally, the cropped codes are converted to the RGB format^4^. The obtained codes are ${\textbf {x}} \in \mathbb {R}^{330 \times 330}$ with symbols’ size $5 \times 5$ elements. Examples of the obtained code is shown in Fig. 5b.

To simulate typical scenario for an unexperienced counterfeiter, the copy fakes were produced based on standard copy machines. The two different copy machines in copy regime “text” were used: *(1)* RICOH MP C307 and *(2)* Samsung CLX-6220FX. The fakes were produced on two types of paper: white paper 80 g/m^2^ and gray paper 80 g/m^2^.

Thus, as it is mentioned in [14], the four fake codes for each original printed code were produced, namely:

- *Fakes #1 white*: made by the copy machine (1) on the white paper.
- *Fakes #1 gray*: made by the copy machine (1) on the gray paper.
- *Fakes #2 white*: made by the copy machine (2) on the white paper.
- *Fakes #2 gray*: made by the copy machine (2) on the gray paper.

To be coherent with the enrolled original printed codes, the acquisition of the produced fakes is performed in the same way using the same mobile phone under the same photo and light settings as for the original printed codes.

In total, the Indigo mobile dataset contains 1800 codes: 300 distinct digital templates, 300 enrolled original printed codes, and 1200 enrolled fake printed codes: 300 originals $\times$ 4 type of fakes.

Examples of the obtained digital, original, and fake codes are shown in Fig. 5. Due to a built-in morphological processing of the Ricoh copy machine, the fakes #1 are more accurate with a dot gain close to the original codes. In the case of the fakes #2, the dot gain is much higher and, as a result, the symbols contain more black ink and look darker. Visually, the difference between the two types of used paper is not evident.

For the empirical evaluation, the Indigo mobile dataset was split into three sub-sets: *training* with 40% of data, *validation* with 10% of data, and 50% of data is used for the *test*. To avoid the bias in the choice of training and test data, each investigated model was trained five times under randomly splitting data between these subsets. Moreover, the following data augmentations were used: *(i)* the rotations on $90^{\circ }$, $180^{\circ }$ and $270^{\circ }$; *(ii)* the gamma correction with variable function $(.)^\gamma$, where $\gamma \in [0.5, 1.2]$ with step 0.1 is the parameter of gamma correction.

## Multi-class supervised classification

### Theoretical analysis

The supervised multi-class classification is chosen as a base-line to validate the authentication efficiency of CDP. The complete availability of fakes at the training stage for the classification gives the defender an information advantage over the attacker. Such a scenario is an ideal case for the defender and the worst case for the attacker. It assumes that, besides the original digital templates $\{{\textbf {t}}_i\}_{i=1}^M$ and the corresponding printed codes $\{{\textbf {x}}_i\}_{i=1}^M$, the defender has an access to the fake codes $\{{\textbf {f}}_i\}_{i=1}^{M_f}$.

From the information-theoretic point of view, the problem of a supervised classifier training given the labeled data $\{{\textbf {y}}_i, {\textbf {c}}_i\}^N_{i=1}$ generated from a joint distribution $p({\textbf {y}}, {\textbf {c}})$^5^ is formulated as a training of a parameterized network $p_{\varvec{\phi }}({\textbf {c}}|{\textbf {y}})$ that is an approximation of $p({\textbf {c}}|{\textbf {y}})$ originating from the chain rule decomposition $p({\textbf {y}}, {\textbf {c}}) = p_{\mathcal {D}}({\textbf {y}}) p({\textbf {c}}|{\textbf {y}})$. The training of the network $p_{\varvec{\phi }}({\textbf {c}}|{\textbf {y}})$ is performed based on the maximization of a mutual information $I_{\varvec{\phi }}({\textbf {Y}};{\textbf {C}})$ between ${\textbf {y}}$ and ${\textbf {c}}$ via $p_{\varvec{\phi }}({\textbf {c}}|{\textbf {y}})$:

1 $$\begin{aligned} \hat{\varvec{\phi }} = \underset{\varvec{\phi }}{\text {argmax}} I_{\varvec{\phi }}({\textbf {Y}};{\textbf {C}}), \end{aligned}$$

that can be rewritten as:

2 $$\begin{aligned} \hat{\varvec{\phi }} = \underset{\varvec{\phi }}{\text {argmin}} {\mathcal L}_{\text {Supervised}}(\varvec{\phi }), \end{aligned}$$

where $${\mathcal L}_{\text {Supervised}}(\varvec{\phi } ) = - I_{\varvec{\phi }}({\textbf {Y}};{\textbf {C}})$$.

As it was shown in [18] the mutual information in (1) can be defined as:

3 $$\begin{aligned} I_{\varvec{\phi }}({\textbf {Y}}; {\textbf {C}})\triangleq & {} \mathbb {E}_{p({\textbf {y}},{\textbf {c}})} \left[ \log \frac{p_{\varvec{\phi }}({\textbf {c}}|{\textbf {y}})}{p_c({\textbf {c}})} \right] \nonumber \\= & {} \underbrace{\mathbb {E}_{p({\textbf {y}},{\textbf {c}})} \left[ \log p_{\varvec{\phi }}({\textbf {c}}|{\textbf {y}}) \right] }_{\mathcal {D}_{{\textrm{c}} \hat{\textrm{c}}}} - \underbrace{\mathbb {E}_{p_c({\textbf {c}})} \left[ \log p_c({\textbf {c}}) \right] }_{= \text {constant}}, \end{aligned}$$

where $H({\textbf {C}}) = -\mathbb {E}_{p_c({\textbf {c}})} \left[ \log p_c(c) \right]$ is the entropy of ${\textbf {c}}$ and it is a constant that does not depend on $\varvec{\phi }$.

Therefore, the optimization problem (2) reduces to:

4 $$\begin{aligned} \hat{\varvec{\phi }} = \underset{\varvec{\phi }}{\text {argmin}} {\mathcal L}_{\text {Supervised}}(\varvec{\phi }) = \underset{\varvec{\phi }}{\text {argmin}} -\mathcal {D}_{{\textrm{c}} \hat{\textrm{c}}}. \end{aligned}$$

#### Remark 1

In practice, the $\mathcal {D}_{{\textrm{c}} \hat{\textrm{c}}}$ term is optimized with respect to the cross-entropy loss.

### Experimental results

The performance of the presented model (4) was empirically evaluated on the Indigo mobile dataset. The supervised multi-class classification is performed in two scenarios: *(1)* multi-class classification and *(2)* binary classification.

#### Multi-class classification

The multi-class supervised classification aims at investigating the performance of the supervised classification scenario, where the model is trained on all classes of the data. Therefore, it corresponds to the case of the informed defender who knows all types of fakes in advance. At the inference stage, three validation scenarios are evaluated:

- 5-class classification: the ability of the model to distinguish all classes of the data, i.e., originals and four types of fakes
- 3-class classification: the ability of the model to distinguish the originals, fakes from the first (fakes #1) and the second (fakes #2) groups
- 2-class classification: the ability of the model to distinguish the originals from all types of fakes considered as a joint class

Due to the relatively small amount of the codes in the Indigo mobile dataset and to avoid the bias in the selection of data for training and testing, the classification model is trained five times on the randomly chosen subset of data.

At the inference stage, the query sample ${\textbf {y}}$, which might be either the original code ${\textbf {x}}$ or one of the fakes ${\textbf {f}}^k$, $k = 1, ..., 4$, is passed through a deterministic classifier $g_{\varvec{\phi }}$ such that $p_{\varvec{\phi }}({\textbf {c}}|{\textbf {y}}) = \delta ({\textbf {c}} - g_{\varvec{\phi }}({\textbf {y}}))$ and $\delta (.)$ denotes the Dirac delta-function or simply ${\textbf {c}} = g_{\varvec{\phi }}({\textbf {y}})$. Each class is encoded as one-hot-encoding with the class $i^{\text {th}}$ represented as ${\textbf {c}}_i = [0, ..., 1, ..., 0]^{\text {T}}$, with “1” in the position of $i^{\text {th}}$. Herewith, $g_{\varvec{\phi }}$ is trained with respect to the term $\mathcal {D}_{{\textrm{c}} \hat{\textrm{c}}}$ in (4). The term $\mathcal {D}_{{\textrm{c}} \hat{\textrm{c}}}$ represents the cross-entropy in this case. The obtained classification error $P_{e} = Pr[\hat{{\textbf {c}}} \ne \textbf{C} | \textbf{C} = {\textbf {c}}]$ is given in Table 1. It is easy to see that the investigated model is capable to authenticate the original codes without mistakes in all considered scenarios.

**Table 1:** The classification error of the supervised multi-class classifier (in %)

Classification type	Originals	Fakes #1 white	Fakes #1 gray	Fakes #2 white	Fakes # 2 gray
2-class^a^	0.00	0.28			
3-class	0.00	0.78		0.35	
5-class	0.00	23.26	21.56	16.88	11.35

^a^$P_e$ corresponds to the $P_{miss}$ for the originals and to the $P_{fa}$ for the fakes

**Fig. 6:**
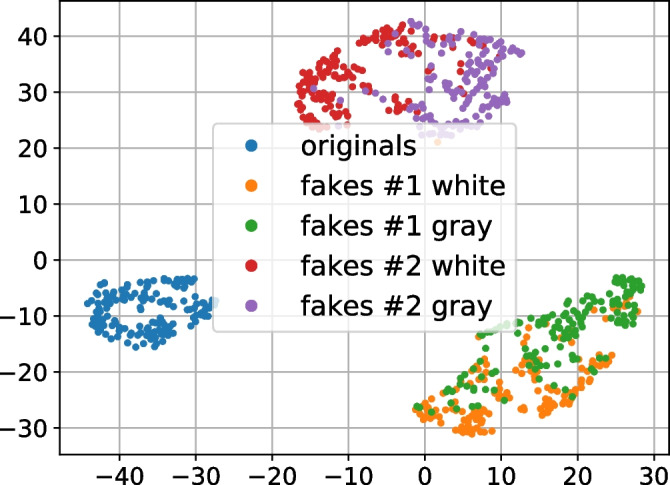
T-SNE of the latent space (the last layer before an activation function) of the supervised classifier trained on originals and all type of fakes. A horizontal axis denotes t-SNE dimension 1 and the t-SNE dimension 2 is on the vertical axis

The classification error about $0.28\%$ in the two classes validation setup (“2-class” label in Table 1) indicates that despite the visual similarity the classifier is capable to distinguish original and fakes with high enough accuracy. From the three classes validation scenario (“3-class” label in Table 1), one can notice that the model confuses more the fakes #1 than fakes #2. The last validation scenario (“5-class” label in Table 1) shows that for both groups of fakes the most difficult is to distinguish between the white and gray paper type of fakes. In addition, in Fig. 6 the t-SNE visualization [19] of the latent space (the last layer before an activation function) of the classifier trained in 5-class classification scenario is illustrated. From that visualization one can easily see the same phenomena: three main classes (originals, fakes #1 and fakes #2) are well separated while the samples printed on the white and gray papers overlap. This indicates that the substrate identification is a difficult problem even for the supervised classifier under the considered imaging setup.

#### Binary classification

The supervised binary classification aims at investigating the influence of the fakes’ type used for the training on the model efficiency at the inference stage. In this respect, the training is performed separately on each type of fakes. Similarly to the multi-class classification scenario, in each case, the model is trained five times on the randomly chosen subset of data to avoid the bias in the training data selection. The difference between the 2-class classification and the considered binary classification consists in the assumption about the fakes available at the training. The 2-class classification assumes that all types of fakes are available at the training stage whereas the binary classification assumes that only one type of fakes is available and the rest fakes are unknown. Obviously, the binary classification is more challenging and the results will highly depend on the type of fakes chosen for training. At the test stage all fakes are present for the classification.

The binary classification accuracy is evaluated with respect to the probability of miss $P_{miss}$ and the probability of false acceptance $P_{fa}$ defined as:

5 $$\begin{aligned} \left\{ \begin{array}{lll} P_{fa} &{} = &{} \text {Pr}\{ g_{\varvec{\phi }}({\textbf {Y}}) = {\textbf {c}}_1 \;|\; \mathcal {H}_0 \}, \\ P_{miss} &{} = &{} \text {Pr}\{ g_{\varvec{\phi }}({\textbf {Y}}) \ne {\textbf {c}}_1 \;|\; \mathcal {H}_1 \}, \end{array} \right. \end{aligned}$$

where ${\textbf {c}}_1 = [1, 0]^{\text {T}}$ denotes a class of original codes, $\mathcal {H}_1$ corresponds to the hypothesis that the query ${\textbf {y}}$ is an original code and $\mathcal {H}_0$ is the hypothesis that the query ${\textbf {y}}$ is a fake code.

**Fig. 7:**
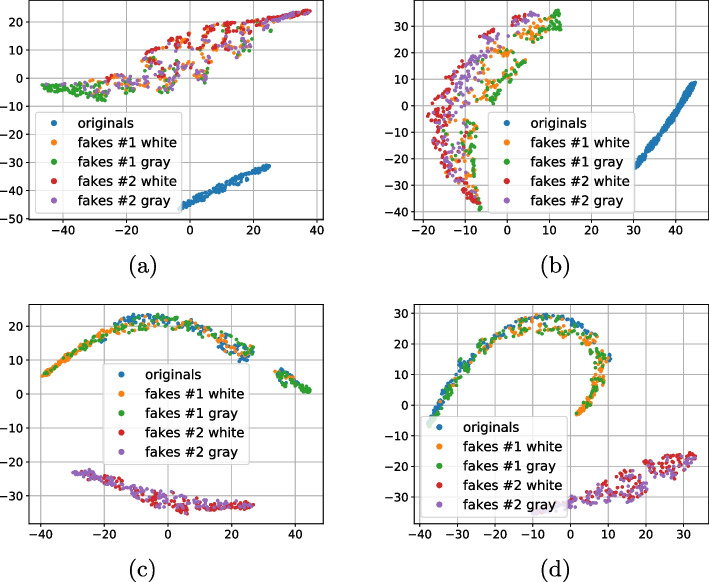
The latent space (the last layer before an activation function) t-SNE visualization of the supervised binary classifier trained on the originals and **a** fakes #1 white, **b** fakes #1 gray, **c** fakes #2 white, **d** fakes #2 gray. A horizontal axis denotes t-SNE dimension 1 and the t-SNE dimension 2 is on the vertical axis

From the obtained results presented in Table 2 one can note that both models trained on the originals and fakes #1 provide high classification accuracy on all type of data, including the fakes #2, unseen during the training. That is expected and can be explained by the fact that, as it is discussed in Section 2.2, the fakes #1 are closer to the originals, while the fakes #2 are the coarser copies of the original codes. In this regard, when the training is performed on the fakes #2, no model is capable to distinguish the originals from the fakes #1, unseen during the training. That is confirmed by the probability of false acceptance close to 100%. Nevertheless, the models are capable to distinguish the originals from the fakes #2 with 100% accuracy. The t-SNE visualization of the latent space of each model illustrated in Fig. 7 confirms these observations. From Fig. 7a and b that present the latent space of models trained on the originals and the fakes #1, one can see the good separability between the originals and fakes while all classes of fakes overlap. The latent space visualization of models trained on the originals and fakes #2 illustrated in Fig. 7c and d shows the overlapping between the originals and the fakes #1 preserving the fakes #2 in well separable cluster.

**Table 2:** The classification error of the supervised binary classifier (in %)^a^

Setup on	Originals	Fakes #1	Fakes #1	Fakes #2	Fakes # 2
	($P_{miss}$)	White ($P_{fa}$)	Gray ($P_{fa}$)	White ($P_{fa}$)	Gray ($P_{fa}$)
Fakes #1 white	0	0	0.14	0	0
Fakes #1 gray	0	0	0	0	0
Fakes #2 white	0	99.43	100	0	0
Fakes # 2 gray	0	99.29	99.86	0	0

^a^Presented binary classification is close to the multi-class classification scenario with 2 classes considered in Section 3.2.1. The difference in the obtained results is related to the presence of all types of fakes during the training in case of multi-class setup and randomly chosen training data

## One-class classification

### Spatial domain data analysis

**Fig. 8:**
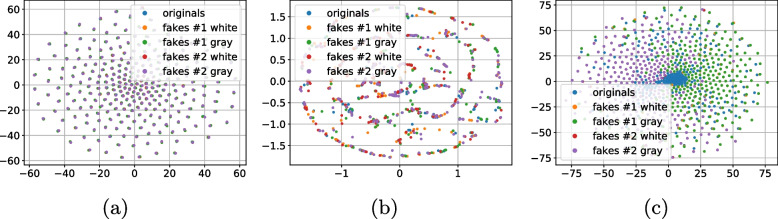
The 2D t-SNE visualization of the original and fake codes in the spatial domain (a horizontal axis denotes t-SNE dimension 1 and the t-SNE dimension 2 is on the vertical axis): **a** presents the direct RGB images’ visualization; **b** is based on the xor difference between the corresponding digital templates and printed codes binarized via a simple thresholding method with an optimal threshold determined individually for each printed code via the Otsu’s method [20]; **c** visualizes the differences between the physical references and the corresponding printed original and fake codes

In Section 3, it is shown that according to results obtained for the Indigo mobile dataset, the original and fake codes are well separable in the latent space of the multi-class supervised classifier (Fig. 6). To answer the question how these data behave in the direct image domain (hereinafter also referred to as a *spatial* domain), the 2D t-SNE visualizations of the data in the spatial domain are shown in Fig. 8.

Figure 8a shows the direct visualization of the RGB images. One can note that the data do not form any clusters corresponding to originals or fakes. Instead, the data are allocated into small groups that are formed by the originals and fakes corresponding to the same digital template. Such a behavior is expectable and is explainable by the data nature.

**Fig. 9:**
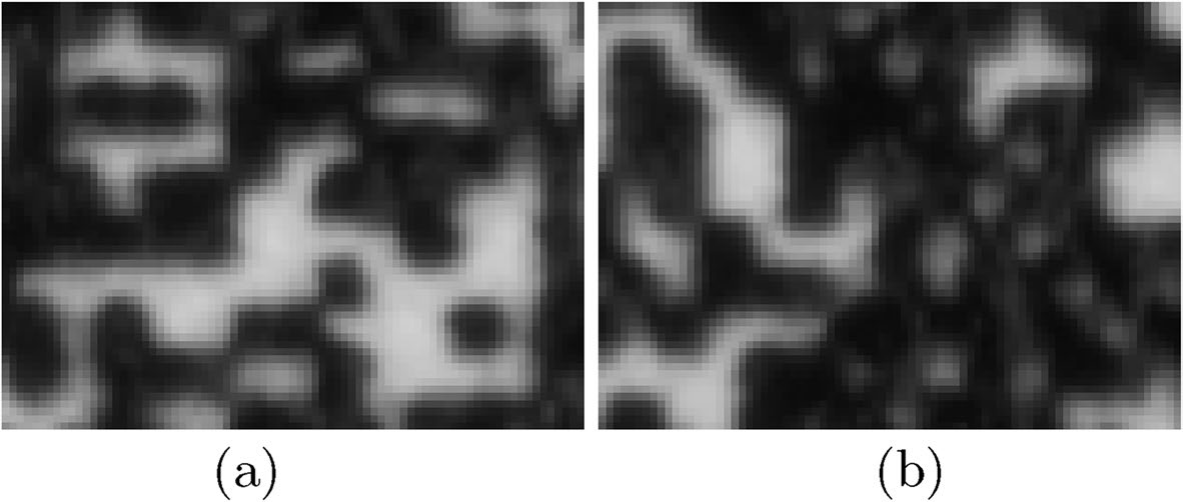
Examples of the dot gain effect: **a** a black symbol surrounded by white symbols increases its size but remains well detectable; **b** a white symbol surrounded by black symbols might disappear under strong dot gain

Figure 8b demonstrates a visualization based on the xor difference between the digital templates and the corresponding printed codes binarized via a simple thresholding method with an optimal threshold determited individually for each printed code via the Otsu’s method [20]. In general, one can observe a kind of rings that consist of the original and fakes but no clusters specific to the data types are observed. These rings are explainable by the fact that both originals and fakes can have bigger or smaller difference with the digital template due to the dot gain in the different group of black and white symbols as shown in Fig. 9: a white symbol surrounded by the black symbols results in a bigger binarization error, while the black symbol surrounded by the white symbols is more likely to survive after binarization.

To better understand the role of the digital templates as a references, the Indigo mobile dataset was specially extended by the printed references (hereinafter also referred to as *physical references*^6^). It is easy to note the central dense cluster formed by the original codes (in blue) and two surrounding clusters from the fakes #1 (mostly on the right-hand side) and fakes #2 (mostly on the left-hand side) from Fig. 8c that illustrates the t-SNE of the differences between the physical reference and the corresponding printed original and fake codes. Despite this, the overall mixing of individual samples from the different classes is quite significant. This indicates that the reliable direct spatial authentication might be complicated.

**Fig. 10:**
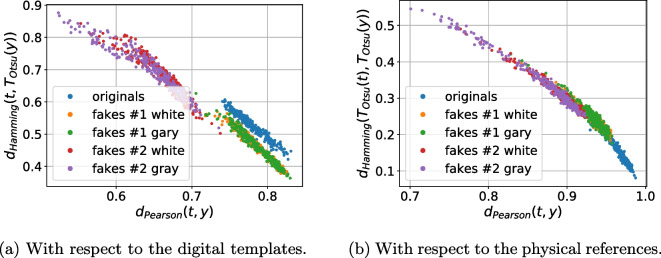
The CDP separability in the 2D space of Pearson correlation (the horizontal axis) and Hamming distance (the vertical axis)

As a next stage we performed the analysis of distances between the references (digital or physical) and the corresponding printed codes (original and fakes) in different metrics: $\ell _1$, $\ell _2$, Pearson correlation and Hamming distance. Whenever needed the binarization is applied via a simple thresholding with an optimal threshold determined individually for each code via the Otsu’s method. The performed analysis demonstrates that besides some rare exceptions, it is impossible to separate the original and fake codes neither with respect to the digital template nor with respect to the physical reference based only on one metric. At the same time, the separability with respect to the two metrics is much better. The best two-metric separability we obtained is based on the Pearson correlation [21] and Hamming distance [22] between the printed codes and the corresponding digital or physical references as shown in Fig. 10a, b. Encouraged by these results, we apply the one-class support vector machines (OC-SVM) [23] in the space of the Pearson correlation and Hamming distance between the printed codes and the corresponding digital or physical references.

To better understand the role of used reference and the influence of color information during the acquisition of black and white codes as opposed to their conversion to only grayscale images, the OC-SVM is applied with respect to four types of training data:

- With respect to the digital templates on:
  - The grayscale original codes ${\textbf {x}}$;
  - The RGB original codes ${\textbf {x}}$.
- With respect to the physical references on:
  - The grayscale original codes ${\textbf {x}}$;
  - The RGB original codes ${\textbf {x}}$.

**Fig. 11:**
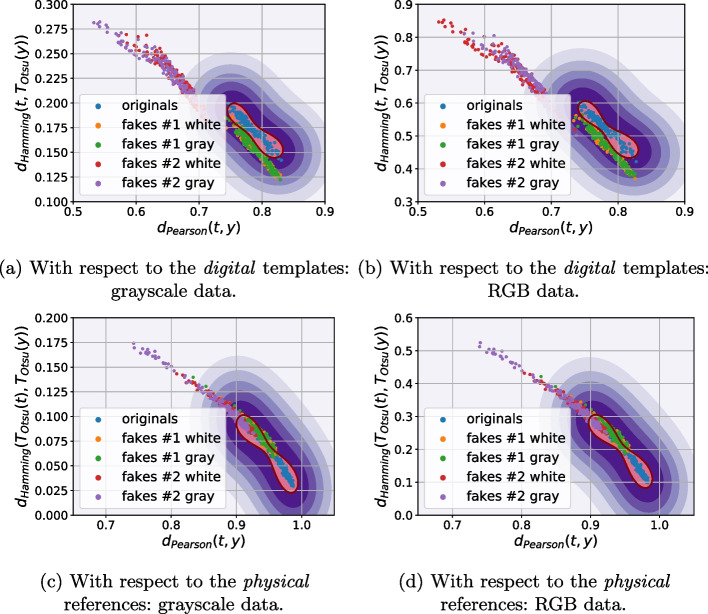
The decision boundaries of OC-SVM trained with respect to the Pearson correlation and Hamming distance between the reference (digital or physical) and the corresponding original printed codes

To avoid the bias in the training data selection, the OC-SVM was trained five times on randomly chosen original printed samples ${\textbf {x}}$ and either digital templates or physical references. The OC-SVM was trained to minimize the $P_{miss}$ on the validation sub-set. The obtained classification error is represented in Table 3. The visualization of the OC-SVM decision boundaries is illustrated in Fig. 11.

**Table 3:** The OC-SVM classification error in spatial domain (in %)^a^

Train on	Originals	Fakes #1	Fakes #1	Fakes #2	Fakes #2
	($P_{miss}$)	White ($P_{fa}$)	Gray ($P_{fa}$)	White ($P_{fa}$)	Gray ($P_{fa}$)
*With respect to the digital templates:*					
- Grayscale ${\textbf {x}}$	3.1	2.54	3.82	0	0
- RGB ${\textbf {x}}$	2.82	2.1	1.4	0	0
*With respect to the physical references:*					
- Grayscale ${\textbf {x}}$	11.44	35.86	40.58	1.72	1.12
- RGB ${\textbf {x}}$	11.16	31.84	39.54	1.44	0.98

^a^The python *OneClassSVM* method from the sklearn package is used with the next training parameters: kernel = “rbf”; gamma = 0.1; nu = 0.03 for the digital templates and nu = 0.1 for the physical references

Analyzing the obtained results, at first, it should be pointed out that the OC-SVM classification error based on the $P_{miss}$ and $P_{fa}$ is relatively high. At the same time, two important conclusions can be done:

- With respect to the chosen metrics, the use of the digital templates is preferable than the printed references.
- Despite the visually grayscale nature of the CDP, the authentication based on codes taken by the mobile phone in color mode is more efficient compared to the grayscale mode due to the fact that the different color channels have different sensitivity and due to the information loss while converting a three-channels color image into a single-channel grayscale one.

### Deep processing domain data analysis

**Fig. 12:**
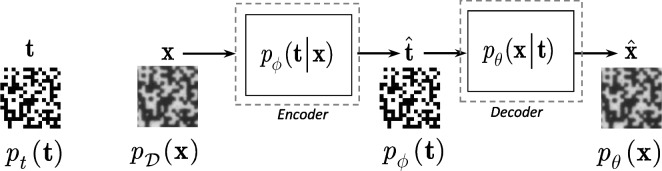
General scheme of a deep model that aims at estimating the digital templates $\hat{{\textbf {t}}}$ from the original printed codes ${\textbf {x}}$ with the following mapping of the estimated digital templates $\hat{{\textbf {t}}}$ back to the printed codes $\hat{{\textbf {x}}}$

To further investigate the authentication performance, we consider an one-class classification based on the features extracted via DNN processing. In a particular case of the CDP authentication, where the reference templates ${\textbf {t}}$ are given, we consider a feature extractor based on a DNN auto-encoder model ${\textbf {x}} \rightarrow \hat{{\textbf {t}}} \rightarrow \hat{{\textbf {x}}}$, where $\hat{{\textbf {t}}}$ is considered as a latent space representation as shown in Fig. 12. The difference with a generic auto-encoder consists in the fact that the latent space is represented by a space of digital templates in contrast to some generic low-dimensional representation in a generic auto-encoder.

The loss-function for the considered feature extracting system is defined as:

6 $$\begin{aligned} {\mathcal L}_{\text {One-class}}(\varvec{\phi }, \varvec{\theta }) = - I_{\varvec{\phi }}({\textbf {X}}; {\textbf {T}}) - \beta I_{\varvec{\phi },\varvec{\theta }}({\textbf {T}}; {\textbf {X}}), \end{aligned}$$

where $\beta$ controls the relative importance of the two objectives.

The first mutual information term $I_{\varvec{\phi }}({\textbf {X}}; {\textbf {T}})$ in (6) controls the mutual information between the estimate of template $\hat{{\textbf {t}}}$ produced from ${\textbf {x}}$ based on the mapper $p_{\varvec{\phi }}({\textbf {t}}|{\textbf {x}})$ and original template ${\textbf {t}}$ and is defined as:

7 $$\begin{aligned} I_{\varvec{\phi }}({\textbf {X}}; {\textbf {T}})= & {} \mathbb {E}_{p({\textbf {x}},{\textbf {t}})} \left[ \log \frac{p({\textbf {x}},{\textbf {t}})}{p_{\mathcal {D}}({\textbf {x}}) {p_t({{\textbf {t}}})}} \right] \nonumber \\= & {} \mathbb {E}_{p({\textbf {x}},{\textbf {t}})} \left[ \log \frac{p_{\mathcal {D}}({\textbf {x}}) p_{\varvec{\phi }}({\textbf {t}}|{\textbf {x}})}{p_{\mathcal {D}}({\textbf {x}}) {p_t({{\textbf {t}}})}} \right] \nonumber \\= & {} \mathbb {E}_{p({\textbf {x}},{\textbf {t}})} \left[ \log \frac{p_{\varvec{\phi }}({\textbf {t}}|{\textbf {x}})}{p_t({\textbf {t}})} \right] . \end{aligned}$$

According to [24], the variational decomposition is applied to decompose (7) into a form suitable for the practical calculations:

8 $$\begin{aligned} I_{\varvec{\phi }}({\textbf {X}}; {\textbf {T}})= & {} \mathbb {E}_{p({\textbf {x}},{\textbf {t}})} \left[ \log \frac{p_{\varvec{\phi }}({\textbf {t}}|{\textbf {x}})}{p_t({\textbf {t}})} \frac{p_{\varvec{\phi }}({\textbf {t}})}{p_{\varvec{\phi }}({\textbf {t}})} \right] \nonumber \\= & {} - \mathbb {E}_{p_t(t)} \left[ \log \frac{p_t({\textbf {t}})}{p_{\varvec{\phi }}({\textbf {t}})} \right] - \mathbb {E}_{p_t({\textbf {t}})} \left[ \log p_{\varvec{\phi }}({\textbf {t}})\right] \nonumber \\{} & {} + \mathbb {E}_{p_{\mathcal {D}}({\textbf {x}})} \left[ \mathbb {E}_{p_{\varvec{\phi }}({\textbf {t}}|{\textbf {x}})} \left[ \log p_{\varvec{\phi }}({\textbf {t}}|{\textbf {x}}) \right] \right] , \end{aligned}$$

where $D_{\textrm{KL}}\left( p_t({\textbf {t}}) \Vert p_{\varvec{\phi }}({\textbf {t}}) \right) = \mathbb {E}_{p_t(t)} \left[ \log \frac{p_t({\textbf {t}})}{p_{\varvec{\phi }}({\textbf {t}})} \right]$ is a Kullback-Leibler divergences between the true $p_t({\textbf {t}})$ and the posterior $p_{\varvec{\phi }}({\textbf {t}})$. $H(p_t({\textbf {t}}), p_{\varvec{\phi }}({\textbf {t}})) = - \mathbb {E}_{p_t({\textbf {t}})} \left[ \log p_{\varvec{\phi }}({\textbf {t}})\right]$ is a cross-entropy.

Taking into account that the cross-entropy $H(p_t({\textbf {t}}), p_{\varvec{\phi }}({\textbf {t}})) \ge 0$, we get $I_{\varvec{\phi }}({\textbf {X}}; {\textbf {T}}) \ge I_{\varvec{\phi }}^L({\textbf {X}}; {\textbf {T}})$, where:

9 $$\begin{aligned} I_{\varvec{\phi }}^L({\textbf {X}}; {\textbf {T}})\triangleq & {} \underbrace{\mathbb {E}_{p_{\mathcal {D}}({\textbf {x}})} \left[ \mathbb {E}_{p_{\varvec{\phi }}({\textbf {t}}|{\textbf {x}})} \left[ \log p_{\varvec{\phi }}({\textbf {t}}|{\textbf {x}}) \right] \right] }_{\mathcal {D}_{\textrm{t} \mathrm{\hat{t}}}} \nonumber \\{} & {} - \underbrace{D_{\textrm{KL}}\left( p_t({\textbf {t}}) \Vert p_{\varvec{\phi }}({\textbf {t}}) \right) }_{\mathcal {D}_{\textrm{t}}}. \end{aligned}$$

The second mutual information term in (6) determined as $I_{\varvec{\phi },\varvec{\theta }}({\textbf {T}};{\textbf {X}}) = \mathbb {E}_{p_{\mathcal {D}}({\textbf {x}})} \left[ \mathbb {E}_{p_{\varvec{\phi }}({\textbf {t}}|{\textbf {x}})} \left[ \log \frac{p_{\varvec{\theta }}({\textbf {x}}|{\textbf {t}})}{p_{\mathcal {D}}({\textbf {x}})} \right] \right]$ can be decomposed and bounded in a way similar to the first term: $I_{\varvec{\phi },\varvec{\theta }}({\textbf {T}};{\textbf {X}}) \ge I^L_{\varvec{\phi }, \varvec{\theta }}({\textbf {T}};{\textbf {X}})$, where:

10 $$\begin{aligned} I^L_{\varvec{\phi },\varvec{\theta }}({\textbf {T}};{\textbf {X}})\triangleq & {} \underbrace{\mathbb {E}_{p_{\mathcal {D}}({\textbf {x}})} \left[ \mathbb {E}_{p_{\varvec{\phi }}({\textbf {t}}|{\textbf {x}})} \left[ \log p_{\varvec{\theta }}({\textbf {x}}| {\textbf {t}}) \right] \right] }_{\mathcal {D}_{{\textrm{x}} \hat{\textrm{x}}}} \nonumber \\{} & {} - \underbrace{D_{\textrm{KL}}\left( p_{\mathcal {D}}({\textbf {x}} ) \Vert p_{\varvec{\theta }}({\textbf {x}})\right) }_{\mathcal {D}_{\textrm{x}}}. \end{aligned}$$

#### Remark 2

The term $\mathcal {D}_{\textrm{t}}$ in (9) and the term $\mathcal {D}_{\textrm{x}}$ in (10) can be implemented based on the density ratio estimation [25]. The terms $\mathcal {D}_{{\textrm{t}} \hat{\textrm{t}}}$ and $\mathcal {D}_{{\textrm{x}} \hat{\textrm{x}}}$ can be defined explicitly using Gaussian or Laplacian priors. In the Gaussian case, one can define $p_{\varvec{\phi }}({\textbf {t}}|{\textbf {x}}) \propto \exp (-\lambda _1\Vert {\textbf {t}} - g_{\varvec{\phi }}({\textbf {x}})\Vert _2)$ and $p_{\varvec{\theta }}({\textbf {x}}|{\textbf {t}}) \propto \exp (-\lambda _2\Vert {\textbf {x}} - f_{\varvec{\theta }}({\textbf {t}})\Vert _2)$ with the scale parameters $\lambda _1$ and $\lambda _2$, which lead to $\ell _2$-norm, and $g_{\varvec{\phi }}({\textbf {x}})$ denotes the encoder and $f_{\varvec{\theta }}$ denotes the decoder. It also corresponds to the model ${\textbf {t}} = g_{\varvec{\phi }}({\textbf {x}}) + {\textbf {e}}_{\text {x}}$ and ${\textbf {x}} = f_{\varvec{\theta }}({\textbf {t}}) + {\textbf {e}}_{\text {t}}$, where ${\textbf {e}}_{\text {x}}$ and ${\textbf {e}}_{\text {t}}$ are the corresponding reconstruction error vectors following the Gaussian pdf.

Thus, Equation (9) reduces to:

11 $$\begin{aligned} I^L_{\varvec{\phi }}({\textbf {X}};{\textbf {T}})= & {} \underbrace{ - \lambda _1 \mathbb {E}_{p_{\mathcal {D}}({\textbf {x}})} \left[ \mathbb {E}_{p_{\varvec{\phi }}({\textbf {t}}|{\textbf {x}})} \left[ \Vert {\textbf {t}} - g_{\varvec{\phi }}({\textbf {x}})\Vert _2 \right] \right] }_{\mathcal {D}_{{\textrm{t}} \hat{\textrm{t}}}}\nonumber \\{} & {} - \underbrace{D_{\textrm{KL}}\left( p_t({\textbf {t}} ) \Vert p_{\varvec{\phi }}({\textbf {t}})\right) }_{\mathcal {D}_{\textrm{t}}}, \end{aligned}$$

and (10) reduces to:

12 $$\begin{aligned} I^L_{\varvec{\phi },\varvec{\theta }}({\textbf {T}};{\textbf {X}})\triangleq & {} \underbrace{- \lambda _2\mathbb {E}_{p_{\mathcal {D}}({\textbf {x}})} \left[ \mathbb {E}_{p_{\varvec{\phi }}({\textbf {t}}|{\textbf {x}})} \left[ \Vert {\textbf {x}} - f_{\varvec{\theta }}({\textbf {t}})\Vert _2 \right] \right] }_{\mathcal {D}_{{\textrm{x}} \hat{\textrm{x}}}}\nonumber \\{} & {} - \underbrace{D_{\textrm{KL}}\left( p_{\mathcal {D}}({\textbf {x}} ) \Vert p_{\varvec{\theta }}({\textbf {x}})\right) }_{\mathcal {D}_{\textrm{x}}}, \end{aligned}$$

**Fig. 13:**
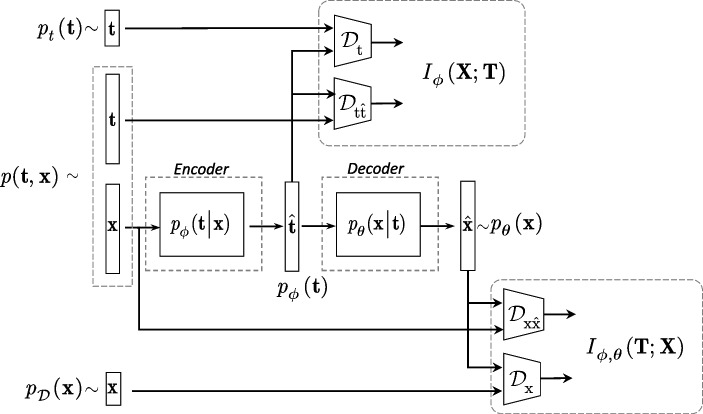
The feature extraction for the one-class classification based on the estimation of the reference templates via $\mathcal {D}_{{\textrm{t}} \hat{\textrm{t}}}$ and $\mathcal {D}_{\textrm{t}}$ and the printed codes via $\mathcal {D}_{{\textrm{x}} \hat{\textrm{x}}}$ and $\mathcal {D}_{\textrm{x}}$ terms

The final optimization problem schematically shown in Fig. 13 is:

13 $$\begin{aligned} ({\hat{\varvec{\phi}}}, \hat{\varvec{\theta }})= & {} \underset{\varvec{\phi }, \varvec{\theta }}{\text {argmin}} {\mathcal L}_{\text {One-class}}^{L}(\varvec{\phi }, \varvec{\theta }) \nonumber \\= & {} \underset{\varvec{\phi }, \varvec{\theta }}{\text {argmin}} - (\mathcal {D}_{{\textrm{t}}\hat{\textrm{t}}} - \mathcal {D}_{\textrm{t}}) - \beta (\mathcal {D}_{{\textrm{x}} \hat{\textrm{x}}} - \mathcal {D}_{\textrm{x}}). \end{aligned}$$

where:

14 $$\begin{aligned} \mathcal {D}_{{\textrm{t}} \hat{\textrm{t}}}\triangleq & {} \mathbb {E}_{p_{\mathcal {D}}({\textbf {x}})} \left[ \mathbb {E}_{p_{\varvec{\phi }}({\textbf {t}}|{\textbf {x}})} \left[ \log p_{\varvec{\phi }}({\textbf {t}}|{\textbf {x}}) \right] \right] ,\nonumber \\ \mathcal {D}_{\textrm{t}}\triangleq & {} D_{\textrm{KL}}\left( p_t({\textbf {t}}) \Vert p_{\varvec{\phi }}({\textbf {t}}) \right) ,\nonumber \\ \mathcal {D}_{{\textrm{x}} \hat{\textrm{x}}}\triangleq & {} \mathbb {E}_{p_{\mathcal {D}}({\textbf {x}})} \left[ \mathbb {E}_{p_{\varvec{\phi }}({\textbf {t}}|{\textbf {x}})} \left[ \log p_{\varvec{\theta }}({\textbf {x}}| {\textbf {t}}) \right] \right] ,\nonumber \\ \mathcal {D}_{\textrm{x}}\triangleq & {} D_{\textrm{KL}}\left( p_{\mathcal {D}}({\textbf {x}}) \Vert p_{\varvec{\theta }}({\textbf {x}})\right) . \end{aligned}$$

In practice, we considered four basic scenarios of features extractors for the one-class classification:

1. The reference templates estimation based on the term $\mathcal {D}_{{\textrm{t}} \hat{\textrm{t}}}$:
  15 $$\begin{aligned} {\mathcal L}_{\text {One-class}}^{1}(\varvec{\phi }, \varvec{\theta }) = -\mathcal {D}_{{\textrm{t}} \hat{\textrm{t}}}. \end{aligned}$$
2. The reference templates estimation based on the terms $\mathcal {D}_{{\textrm{t}} \hat{\textrm{t}}}$ and $\mathcal {D}_{\textrm{t}}$:
  16 $$\begin{aligned} {\mathcal L}_{\text {One-class}}^2(\varvec{\phi }, \varvec{\theta }) = -\mathcal {D}_{{\textrm{t}} \hat{\textrm{t}}} + \mathcal {D}_{\textrm{t}}. \end{aligned}$$
3. The estimation of the reference templates and the printed codes based on terms $\mathcal {D}_{\textrm{t} \mathrm{\hat{t}}}$ and $\mathcal {D}_{\textrm{x} \mathrm{\hat{x}}}$:
  17 $$\begin{aligned} {\mathcal L}_{\text {One-class}}^{3}(\varvec{\phi }, \varvec{\theta }) = -\mathcal {D}_{{\textrm{t}} \mathrm{\hat{t}}} - \beta \mathcal {D}_{\textrm{x} \mathrm{\hat{x}}}. \end{aligned}$$
4. The estimation of the reference templates and the printed codes based on terms $\mathcal {D}_{{\textrm{t}} \hat{\textrm{t}}}$, $\mathcal {D}_{\textrm{t}}$, $\mathcal {D}_{{\textrm{x}} \hat{\textrm{x}}}$ and $\mathcal {D}_{\textrm{x}}$:
  18 $$\begin{aligned} {\mathcal L}_{\text {One-class}}^{4}(\varvec{\phi }, \varvec{\theta }) = -\mathcal {D}_{{\textrm{t}} \hat{\textrm{t}}} + \mathcal {D}_{\textrm{t}} - \beta \mathcal {D}_{\textrm{x} \mathrm{\hat{x}}} + \beta \mathcal {D}_{\textrm{x}}. \end{aligned}$$

In general case, to be comparable with the one-class classification in the spatial domain discussed in Section 4.1, the one-class classification model based on the OC-SVM is used.

**Fig. 14:**
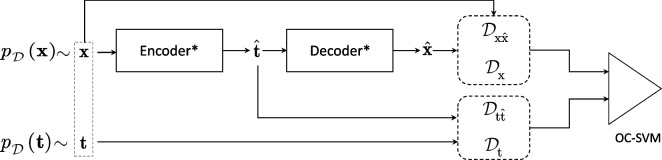
The one-class classification training procedure: the encoder and decoder parts of the auto-encoder model shown in Fig. 13 are pre-trained and fixed (as indicated by a “*”); the OC-SVM is trained on the outputs of $\mathcal {D}_{{\textrm{t}} \hat{\textrm{t}}}$ and $\mathcal {D}_{\textrm{t}}$ terms that are the results of $I^L_{\varvec{\phi }}({\textbf {X}};{\textbf {T}})$ decomposition and the $\mathcal {D}_{{\textrm{x}} \hat{\textrm{x}}}$ and $\mathcal {D}_{\textrm{x}}$ terms that are the results of $I^L_{\varvec{\phi },\varvec{\theta }}({\textbf {T}};{\textbf {X}})$ decomposition

The OC-SVM training procedure shown in Fig. 14 uses the pre-trained and fixed encoder and decoder parts of the auto-encoder model that serves as a features extractor. As an input, the OC-SVM might take different combinations of outputs of four main terms: $\mathcal {D}_{{\textrm{t}} \hat{\textrm{t}}}$, $\mathcal {D}_{\textrm{t}}$, $\mathcal {D}_{{\textrm{x}} \hat{\textrm{x}}}$ and $\mathcal {D}_{\textrm{x}}$. The exact scenarios are discussed in Sections 4.3.1, 4.3.2, 4.3.3, and 4.3.4 below.

### Experimental results

#### First scenario

The optimization problem based on ${\mathcal L}_{\text {One-class}}^1(\varvec{\phi }, \varvec{\theta }) = -\mathcal {D}_{\textrm{t} \mathrm{\hat{t}}}$ aims at producing an accurate estimation $\hat{{\textbf {t}}}$ of the corresponding binary digital template ${\textbf {t}}$ for each input printed original code ${\textbf {x}}$. Taking into account that due to the nature of the used trained model the output estimation is real valued but not binary, at the inference stage, to measure the Hamming distance the final estimation $\hat{{\textbf {t}}}$ is obtained by the thresholding with a threshold 0.5.

**Fig. 15:**
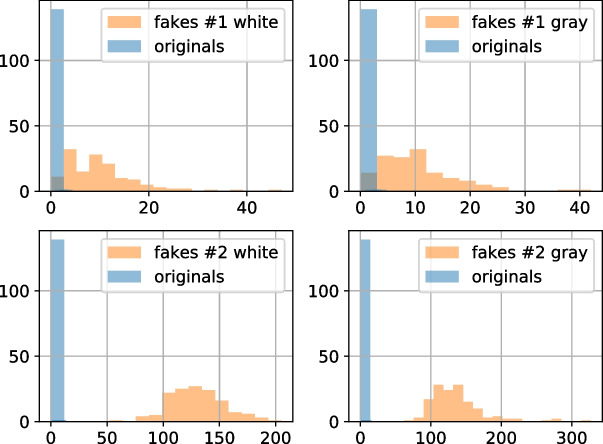
The first scenario results’ visualization: the histogram of symbol-wise Hamming distance (horizontal axis) between the original digital templates ${\textbf {t}}$ and the corresponding estimations $\hat{{\textbf {t}}}$ obtained via the encoder model trained with respect to the term $\mathcal {D}_{{\textrm{t}} \hat{\textrm{t}}}$

Figure 15 illustrates the distributions of the symbol-wise Hamming distance between the original digital templates ${\textbf {t}}$ and the corresponding estimations $\hat{{\textbf {t}}}$ obtained from the printed original and fake codes. Taking into account that the extracted feature vector consists only of one value, the OC-SVM is not used and the classification is performed based on the decision rule:

19 $$\begin{aligned} \left\{ \begin{array}{lll} P_{fa} &{} = &{} \text {Pr}\{d_{\text {Hamming}}({\textbf {t}}, \hat{{\textbf {t}}}) \le \gamma _1 \;|\; \mathcal {H}_0 \}, \\ P_{miss} &{} = &{} \text {Pr}\{ d_{\text {Hamming}}({\textbf {t}},\hat{{\textbf {t}}})> \gamma _1 \;|\; \mathcal {H}_1\}, \end{array} \right. \end{aligned}$$

where $P_{miss}$ is a probability of miss and $P_{fa}$ is probability of false acceptance. The hypothesis $\mathcal {H}_0$ corresponds to the hypothesis that the input code is fake and the $\mathcal {H}_1$ corresponds to the hypothesis that the input code is original. Aiming to have $P_{miss} = 0$, the decision threshold $\gamma _1$ is determined on the validation sub-set to be equal to 2. The obtained classification error is given in Table 4.

**Table 4:** The OC-SVM classification error in deep processing domain (in %)^a^

Model	Originals	Fakes #1	Fakes #1	Fakes #2	Fakes #2
	($P_{miss}$)	White ($P_{fa}$)	Gray ($P_{fa}$)	White ($P_{fa}$)	Gray ($P_{fa}$)
*Based on the Eq.* (19)					
$\mathcal {L}_{\text {One-class}}^1\;$:	0	6.38	8.23	0	0
$$- \mathcal {D}_{{\textrm{t}} \hat{\textrm{t}}}$$					
${\mathcal L}_{\text {One-class}}^2\;$:	0	6.81	7.09	0	0
$$- \mathcal {D}_{{\textrm{t}} \hat{\textrm{t}}} + \mathcal {D}_{\textrm{t}}$$					
${\mathcal L}_{\text {One-class}}^3\;$:	0	1.56	0.99	0	0
$$- \mathcal {D}_{{\textrm{t}} \hat{\textrm{t}}} - \beta \mathcal {D}_{{\textrm{x}} \hat{\textrm{x}}}$$					
${\mathcal L}_{\text {One-class}}^4\;$:	0	2.41	2.13	0	0
$$- \mathcal {D}_{{\textrm{t}} \hat{\textrm{t}}} + \mathcal {D}_{\textrm{t}} - \beta \mathcal {D}_{{\textrm{x}} \hat{\textrm{x}}} + \beta \mathcal {D}_{\textrm{x}}$$					
*Based on the Eq.* (20)					
${\mathcal L}_{\text {One-class}}^3\;$:	0	0.28	0	0	0
$$- \mathcal {D}_{{\textrm{t}} \hat{\textrm{t}}} - \beta \mathcal {D}_{{\textrm{x}} \hat{\textrm{x}}}$$					
${\mathcal L}_{\text {One-class}}^4\;$:	0.57	0	0.14	0	0
$$- \mathcal {D}_{\textrm{t} \mathrm{\hat{t}}} + \mathcal {D}_{\textrm{t}} - \beta \mathcal {D}_{{\textrm{x}} \hat{\textrm{x}}} + \beta \mathcal {D}_{\textrm{x}}$$					
*Based on the OC-SVM*					
${\mathcal L}_{\text {One-class}}^3\;$:	0.28	0	0	0	0
$$- \mathcal {D}_{{\textrm{t}} \hat{\textrm{t}}} - \beta \mathcal {D}_{{\textrm{x}} \hat{\textrm{x}}}$$					
${\mathcal L}_{\text {One-class}}^4\;$:	0.14	0	0	0	0
$$- \mathcal {D}_{{\textrm{t}} \hat{\textrm{t}}} + \mathcal {D}_{\textrm{t}} - \beta \mathcal {D}_{{\textrm{x}} \hat{\textrm{x}}} + \beta \mathcal {D}_{\textrm{x}}$$					

^a^The python *OneClassSVM* method from the sklearn package is used with the following training parameters: kernel = “rbf”; gamma = 0.1; nu = 0.0005

According to the obtained results, the one-class classification based on the encoder model trained with respect to the $\mathcal {D}_{{\textrm{t}} \hat{\textrm{t}}}$ term as shown in Fig. 13 allows to distinguish the originals and the fakes #2 with 100% accuracy. The obtained $P_{miss}$ and $P_{fa}$ are confirmed by the distribution of the Hamming distance shown in Fig. 15. In case of the fakes #1, the corresponding distributions overlap and the $P_{fa}$ is about 6 - 8%.

#### Second scenario

The optimization problem based on ${\mathcal L}_{\text {One-class}}^2(\varvec{\phi }, \varvec{\theta }) = -\mathcal {D}_{{\textrm{t}} \hat{\textrm{t}}} + \mathcal {D}_{\textrm{t}}$ is an extension of the scenario 4.3.1 with the discriminator part $\mathcal {D}_{\textrm{t}}$ that aims to distinguish between the distribution of original digital templates and its corresponding estimate.

**Fig. 16:**
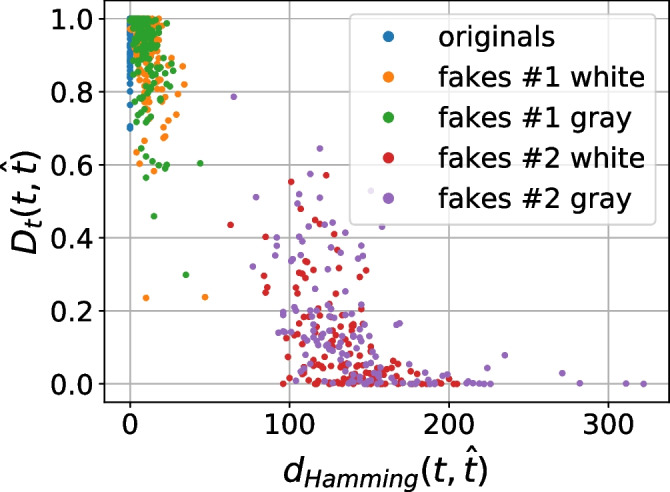
The second scenario results’ visualization: the 2D distribution of *(i)* the symbol-wise Hamming distance between the original digital templates ${\textbf {t}}$ and the corresponding estimations $\hat{{\textbf {t}}}$ obtained via the encoder model trained with respect to the $\mathcal {D}_{{\textrm{t}} \hat{\textrm{t}}}$ term and *(ii)* the corresponding responses of the discriminator model trained with respect to the $\mathcal {D}_{\textrm{t}}$ term

Figure 16 presents the 2D distribution of *(i)* the symbol-wise Hamming distance between the original digital templates ${\textbf {t}}$ and the corresponding estimations $\hat{{\textbf {t}}}$ obtained based on the encoder model trained with respect to the $\mathcal {D}_{{\textrm{t}} \hat{\textrm{t}}}$ term and *(ii)* the corresponding responses of the discriminator trained with respect to the $\mathcal {D}_{\textrm{t}}$ term as shown in Fig. 13. It is easy to see that the obtained results are very close to those in Fig. 15 with respect to the Hamming distance, namely, the results for the original codes are close to zero and overlap with the fakes #1, while the fakes #2 are well separable. With respect to the $\mathcal {D}_{\textrm{t}}$ discriminator decision the situation is similar, namely, the fakes #2 are well separable by the decision ratio smaller then 0.5 - 0.6. At the same time, for the the fakes #1 the decision ratio is bigger than 0.7 - 0.8 as well as for the originals.

The obtained authentication error based on the $P_{miss}$ and $P_{fa}$ calculated with respect to the decision rule (19) and given in Table 4 shows that the regularization via the discriminator $\mathcal {D}_{\textrm{t}}$ does not have any significant influence and does not allow to improve the authentication accuracy.

#### Third scenario

In the third scenario ${\mathcal L}_{\text {One-class}}^3(\varvec{\phi }, \varvec{\theta }) = -\mathcal {D}_{{\textrm{t}} \hat{\textrm{t}}} - \beta \mathcal {D}_{{\textrm{x}} \hat{\textrm{x}}}$, the term $\mathcal {D}_{{\textrm{x}} \hat{\textrm{x}}}$ is in charge of the printed codes reconstruction and plays a role of a learnable regularization.

**Fig. 17:**
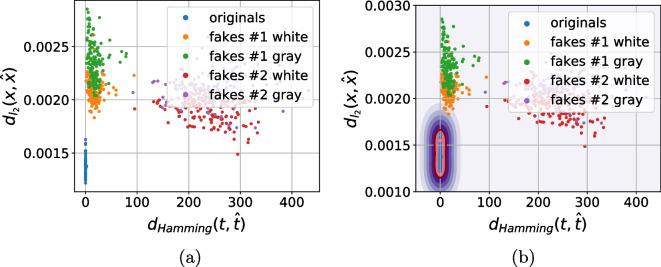
The third scenario results’ visualization: **a** the distribution of *(i)* the symbol-wise Hamming distance between the digital templates and its corresponding estimations via the encoder model trained with respect to the $\mathcal {D}_{{\textrm{t}} \hat{\textrm{t}}}$ term and *(ii)* the $\ell _2$ distance between the printed codes and its corresponding reconstructions by the decoder model trained with respect to the $\mathcal {D}_{{\textrm{x}} \hat{\textrm{x}}}$ term; **b** the OC-SVM decision boundaries

Figure 17a demonstrates the obtained distribution of two metrics: *(i)* the symbol-wise Hamming distance introduced in the Section 4.3.1 and *(ii)* the $\ell _2$ error between the printed codes and the corresponding reconstructions obtained as an output of the decoder model trained with respect to the $\mathcal {D}_{{\textrm{x}} \hat{\textrm{x}}}$ term as shown in Fig. 13 without any additional post-processing.

The obtained authentication results based on the decision rule (19) are given in Table 4. It is easy to see that the learnable regularization via $\mathcal {D}_{{\textrm{x}} \hat{\textrm{x}}}$ term preserves the $P_{miss}$ and $P_{fa}$ on the fakes #2 to be zero, similar to the previous scenarios. At the same time, it allows to decrease the $P_{fa}$ for the fakes #1 from 7% till 1-1.6%. Additionally, Table 4 presents the authentication results obtained based on the two metrics decision rule:

20 $$\begin{aligned} \left\{ \begin{array}{llr} P_{fa} &{} = &{} \text {Pr}\{d_{\text {Hamming}}({\textbf {t}}, \hat{{\textbf {t}}}) \le \gamma _1 \; \& \\ &{} &{} d_{\ell _2}({\textbf {x}}, \hat{{\textbf {x}}}) \le \gamma _2 \;|\; \mathcal {H}_0 \} \\ P_{miss} &{} = &{} \text {Pr}\{ d_{\text {Hamming}}({\textbf {t}}, \hat{{\textbf {t}}})> \gamma _1 \; \& \\ &{} &{} d_{\ell _2}({\textbf {x}}, \hat{{\textbf {x}}}) > \gamma _2 \;|\; \mathcal {H}_1\}, \end{array} \right. \end{aligned}$$

that allows to significantly reduce the $P_{fa}$ for the fakes #1 to about 0.28%. Aiming to have the $P_{miss} = 0$, the decision constant $\gamma _2$ is determined on the validation sub-set to be equal 0.0017 and $\gamma _1$ equals to 2.

In addition, Table 4 includes the results of OC-SVM trained with respect to the metrics under investigation (the symbol-wise Hamming distance between the digital templates and its corresponding estimations via the encoder model trained with respect to the $\mathcal {D}_{{\textrm{t}} \hat{\textrm{t}}}$ term and the $\ell _2$ distance between the printed codes and its corresponding reconstructions by the decoder model trained with respect to the $\mathcal {D}_{{\textrm{x}} \hat{\textrm{x}}}$ term). The OC-SVM is trained only on the train sub-set of the original printed codes ${\textbf {x}}$ and its corresponding templates ${\textbf {t}}$. The example of OC-SVM decision boundaries is illustrated in Fig. 17b. The OC-SVM reduces $P_{fa}$ to 0% for all types of fakes. However, $P_{miss}$ increases to about 0.28% in contrast to the previously obtained results with $P_{miss} = 0\%$.

#### Fourth scenario

**Fig. 18:**
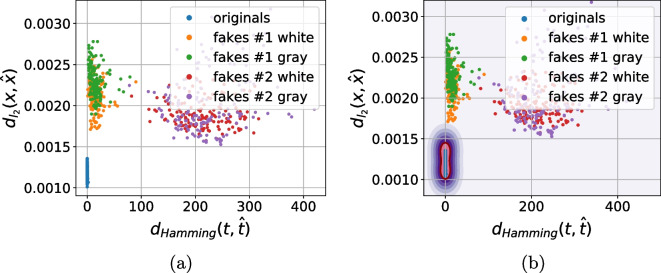
The fourth scenario results’ visualization: **a** the distribution of *(i)* the symbol-wise Hamming distance between the digital templates and its corresponding estimations via the encoder model trained with respect to the $\mathcal {D}_{{\textrm{t}} \hat{\textrm{t}}}$ term and *(ii)* the $\ell _2$ distance between the printed codes and its corresponding reconstructions by the decoder model trained with respect to the $\mathcal {D}_{{\textrm{x}} \hat{\textrm{x}}}$ term; **b** the OC-SVM decision boundaries

The last considered scenario ${\mathcal L}_{\text {One-class}}^4(\varvec{\phi }, \varvec{\theta }) = -\mathcal {D}_{{\textrm{t}} \hat{\textrm{t}}} + \mathcal {D}_{\textrm{t}} - \beta \mathcal {D}_{{\textrm{x}} \hat{\textrm{x}}} + \beta \mathcal {D}_{\textrm{x}}$ includes four terms: the main term $\mathcal {D}_{{\textrm{t}} \hat{\textrm{t}}}$, the discriminator $\mathcal {D}_{\textrm{t}}$ on the digital template estimation space, the printed code reconstruction space regularization $\mathcal {D}_{{\textrm{x}} \hat{\textrm{x}}}$ and the discriminator $\mathcal {D}_{\textrm{x}}$. Similarly to the third scenario, the OC-SVM is trained with respect to the two features: *(i)* the symbol-wise Hamming distance between the original digital templates and their estimations and *(ii)* the $\ell _2$ distance between the printed codes and their reconstructions. A visual representation of the jount distribution of these metrics is shown in Fig. 18a. Table 4 includes the obtained one-class classification error based on three criteria: the decision rules (19) and (20) and the OC-SVM. The example of OC-SVM decision boundaries is illustrated in Fig. 18b.

From the obtained results, one can note that in terms of decision rule (19), the regularization via $\mathcal {D}_{\textrm{t}}$ and $\mathcal {D}_{\textrm{x}}$ discriminators is counter-productive and makes the classification error bigger in comparison with the third scenario. In case of the decision rule (20), the regularization leads to a significant increase of $P_{miss}$. At the same time, the OC-SVM allows to decrease $P_{miss}$ in two times, from 0.28% to 0.14% preserving $P_{fa}$ equals to zero for all types of fakes.

In summary, it should be pointed out that despite the great performance of the fourth scenario’s model its complexity is times higher compared with the other considered scenarios. The execution time complexity in hours per 100 training epochs is given in Table 5 for each scenario.

**Table 5:** Execution time (hours) per 100 epochs on one NVIDIA GPU with a learning rate 1e−4 for the considered scenarios

Model	Execution time, hours
${\mathcal L}_{\text {One-class}}^1: -\mathcal {D}_{{\textrm{t}} \hat{\textrm{t}}}$	2.78–3.05
${\mathcal L}_{\text {One-class}}^2: -\mathcal {D}_{{\textrm{t}} \hat{\textrm{t}}} + \mathcal {D}_{\textrm{t}}$	5.12–5.25
${\mathcal L}_{\text {One-class}}^3: -\mathcal {D}_{{\textrm{t}} \hat{\textrm{t}}} - \beta \mathcal {D}_{{\textrm{x}} \hat{\textrm{x}}}$	5.56–5.83
${\mathcal L}_{\text {One-class}}^4: -\mathcal {D}_{\textrm{t} \mathrm{\hat{t}}} + \mathcal {D}_{\textrm{t}} - \beta \mathcal {D}_{{\textrm{x}} \hat{\textrm{x}}} + \beta \mathcal {D}_{\textrm{x}}$	11.11–11.39

## Conclusion

In this work, we investigate the authentication aspects of modern CDP with respect to the typical hand-crafted copy fakes. To simulate the real-life conditions, we created the Indigo mobile dataset of CDP printed on the industrial printer and enrolled it via the mobile phone under regular light conditions.

The performed analysis of the multi-class supervised classification of CDP reveals two important observations:

- In the general case, the model trained in a supervised way is capable to distinguish with a high accuracy the original CDP from the fakes produced on modern copy machines, which use built-in smart morphological processing enhancing image quality and reducing the dot gain for further reproduction.
- The quality of the fakes used for the training plays a very important role. The superior quality fakes closer to the original codes are of preference for the training and allow the model to authenticate the inferior quality fakes, even when the model does not see them during the training. In contrast, if the classifier is trained on the inferior quality fakes, then it is not capable to authenticate the superior quality fakes.

The performed analysis of CDP authentication based on the one-class classification shows that:

- In view of the great similarity between the original and fake codes, the authentication in the spatial domain *(i)* is difficult with respect to the finding of right metrics and *(ii)* is not reliable enough due to the high overlapping between the classes.
- The authentication with respect to the digital templates is more efficient compared to the authentication with respect to the physical references.
- Despite the original black-and-white nature of the CDP, the authentication based on codes taken by the mobile phone in color mode is more efficient compared to the grayscale mode.
- The authentication with respect to the DNN estimation of the digital templates and printed codes reconstruction is more efficient than the direct authentication with respect to the digital and printed codes in spatial domain.

The main disadvantage of the DNN-based models is its high training complexity compared to the direct authentication in spatial domain. At the same time, at the inference stage, the trained models are equivalent in terms of authentication complexity to the authentication in spatial domain.

Besides the impressive performance of the one-class classification on real samples and mobile phone verification, it should be pointed out that the above analysis is done with respect to the typical HC copy attacks. In view of the widespread use of the ML technologies, the question about the robustness to the ML attacks is an important problem that we aim at investigating in our future work.

## Data Availability

The datasets generated and analyzed during the current study are available at http://sip.unige.ch/projects/snf-it-dis/datasets/indigo-mobile.

## Abbreviations

CDP: Copy detection patterns
PGC: Printable graphical codes
CCD: Charged-couple device
CMOS: Complementary metal-oxide-semiconductor
QR code: Quick response code
2LQR: Two level QR codes
W-QR: Watermarked QR codes
SVM: Support vector machine
OC-SVM: One-class support vector machine
DNN: Deep neural networks
$D_{\textrm{KL}}$: Kullback-Leibler divergences
HC: Hand-crafted
ML: Machine learning

## Footnotes

1 To ensure accurate symbol representation, each printed symbol should be represented by at least $3 \times 3$ pixels. Taking into account the difference between the industrial printing resolution (about 812 dpi) and the average resolution of the mobile phones (about 600–900 ppi) especially in the development countries, where the problem of counterfeiting is particularly important, one can estimate the symbol size from about $4 \times 4$ till $5 \times 5$ pixels.

2 It should be pointed out that the native printing resolution of HP Indigo 5500 DS is 812.8 dpi. The impact of printing resolution and the symbol size is a subject of our ongoing research.

3 https://apps.apple.com/us/app/adobe-lightroom-photo-editor/id878783582

4 https://docs.opencv.org/4.5.2/d8/d01/group__imgproc__color__conversions.html#ga397ae87e1288a81d2363b61574eb8cab

5 ${\textbf {y}}$ might be either the original code ${\textbf {x}}$ or one of the fakes ${\textbf {f}}^k$, $k = 1, ..., 4$.

6 The physical references correspond to the original codes acquired for the second time on the same equipment as the first case scenario. It assumes the probable presence of small geometrical (rotation) and illumination deviations between the original codes and corresponding physical references.
